# Low-Volume Intense Exercise Elicits Post-exercise Hypotension and Subsequent Hypervolemia, Irrespective of Which Limbs Are Exercised

**DOI:** 10.3389/fphys.2016.00199

**Published:** 2016-05-31

**Authors:** Matthew J. Graham, Samuel J. E. Lucas, Monique E. Francois, Stasinos Stavrianeas, Evelyn B. Parr, Kate N. Thomas, James D. Cotter

**Affiliations:** ^1^School of Physical Education, Sport and Exercise Sciences, University of OtagoDunedin, New Zealand; ^2^Department of Physiology, University of OtagoDunedin, New Zealand; ^3^School of Sport, Exercise and Rehabilitation Sciences, University of BirminghamBirmingham, UK; ^4^Department of Exercise Science, Willamette UniversityOregon, USA; ^5^Department of Surgical Sciences, Dunedin School of Medicine, University of OtagoDunedin, New Zealand

**Keywords:** blood pressure, exercise training, plasma volume, HIIT, sprint exercise, arm exercise

## Abstract

**Introduction:** Exercise reduces arterial and central venous blood pressures during recovery, which contributes to its valuable anti-hypertensive effects and to facilitating hypervolemia. Repeated sprint exercise potently improves metabolic function, but its cardiovascular effects (esp. hematological) are less well-characterized, as are effects of exercising upper versus lower limbs. The purposes of this study were to identify the acute (<24 h) profiles of arterial blood pressure and blood volume for (i) sprint intervals versus endurance exercise, and (ii) sprint intervals using arms versus legs.

**Methods:** Twelve untrained males completed three cycling exercise trials; 50-min endurance (legs), and 5^*^30-s intervals using legs or arms, in randomized and counterbalanced sequence, at a standardized time of day with at least 8 days between trials. Arterial pressure, hemoglobin concentration and hematocrit were measured before, during and across 22 h after exercise, the first 3 h of which were seated rest.

**Results:** The post-exercise hypotensive response was larger after leg intervals than endurance (AUC: 7540 ± 3853 vs. 3897 ± 2757 mm Hg·min, *p* = 0.049, 95% CI: 20 to 6764), whereas exercising different limbs elicited similar hypotension (arms: 6420 ± 3947 mm Hg·min, *p* = 0.48, CI: −1261 to 3896). In contrast, arterial pressure at 22 h was reduced after endurance but not after leg intervals (−8 ± 8 vs. 0 ± 7 mm Hg, *p* = 0.04, CI: 7 ± 7) or reliably after arm intervals (−4 ± 8 mm Hg, *p* = 0.18 vs. leg intervals). Regardless, plasma volume expansion at 22 h was similar between leg intervals and endurance (both +5 ± 5%; CI: −5 to 5%) and between leg and arm intervals (arms: +5 ± 7%, CI: −8 to 5%).

**Conclusions:** These results emphasize the relative importance of central and/or systemic factors in post-exercise hypotension, and indicate that markedly diverse exercise profiles can induce substantive hypotension and subsequent hypervolemia. At least for endurance exercise, this hypervolemia may not depend on the volume of post-exercise hypotension. Finally, endurance exercise led to reduced blood pressure the following day, but sprint interval exercise did not.

## Introduction

Good cardiovascular function is important for health and for physical capabilities. A high blood volume and low resting blood pressure are both integral aspects of good cardiovascular function, and both are sensitive to endurance exercise training (Sawka et al., 2000). A principal mechanism by which blood volume increases following exercise appears to be through post-exercise hypotension (PEH) (Nagashima et al., 1999; Hayes et al., 2000). PEH is caused largely by a decreased vascular resistance, which is mediated by both central and peripheral factors and interactions between them—for example, by input from muscle afferents acting centrally to downregulate sympathetic control of peripheral resistance, and also impairing transmission of norepinephrine from sympathetic nerves within the resistance vessels (Halliwill et al., 2013). While such mechanisms have been resolved recently, some aspects of PEH remain less clear, including the dose-vs.-response relation of PEH to the ensuing expansion of plasma volume, and the impact of different exercise parameters (i.e., exercise duration, intensity, and esp. type) on both of these responses. Each exercise parameter will likely have characteristic effects on central and peripheral mediators of PEH (e.g., higher intensity of exercise disproportionately increases sympathetic activation but also humoral mediators of vasodilation and its attenuation), so they could be expected to have different effects on both PEH and exercise-induced hypervolemia.

Whether exercise intensity affects the magnitude of PEH seems unresolved (see: Eicher et al., 2010; Lacombe et al., 2011; Halliwill et al., 2013). For example, sustainable exercise performed at higher intensity (75 vs. 50 or 30% $\overset{\cdot }{\text{V}}$O_2_ max) elicits larger and longer PEH (Forjaz et al., 2004), which may not be attributable to the higher volume of exercise because incremental exercise to 100% $\overset{\cdot }{\text{V}}$O_2_ max has also been found to produce a larger PEH than did 30 min of exercise at 40 or 60% $\overset{\cdot }{\text{V}}$O_2_ max, while they had equivalent effect (Eicher et al., 2010). Whereas, repeated bouts of unsustainable exercise (usually ≥85% $\overset{\cdot }{\text{V}}$O_2_ max), i.e., high-intensity interval exercise, have been shown to induce PEH equivalent to that following typically-sustainable exercise intensities (~60–65% $\overset{\cdot }{\text{V}}$O_2_ max), both when matched for exercise volume (Lacombe et al., 2011), and when performed as maximal-effort sprint intervals and thus involving much lower volume (Rossow et al., 2010). The lack of consistency in findings seems unatttributable to individual factors such as aerobic fitness or resting blood pressure (Lacombe et al., 2011; Halliwill et al., 2013). Nonetheless, PEH facilitates post-exercise plasma restoration (Hayes et al., 2000; Jones et al., 2007) and stimulates hypervolemia (Nagashima et al., 1999, 2000), but the hypervolemic effects of sprint intervals remain largely unknown. This lack of hematological information is in contrast to the wealth of findings showing rapid and marked improvements in several metabolic functions following sprint interval training (Gibala et al., 2006; Burgomaster et al., 2008; Gibala and Mcgee, 2008), and limited knowledge on vascular effects (Rakobowchuk et al., 2008; Whyte et al., 2010). A more complete understanding of the physiological effects of high-intensity interval training, including sprint interval training, is required for practical reasons, i.e., because a minority of people obtain their currently-recommended doses of sustainable exercise per day for health (SPARC, 2008; Tucker et al., 2011). If similar cardiovascular benefits can also be obtained from brief intervals, this would provide more flexibility and variety to exercise prescription, at least for healthy and younger individuals.

The extent to which PEH and expansion of blood volume are stimulated by the size and/or gravitational dependency of the vasculature used in the preceding exercise bout have seldom been examined, and may be an important factor for driving PEH (Miles et al., 1983; de Almeida et al., 2010). The differences in the size and location of the muscle mass used between upper- and lower-body exercise provides one means to investigate whether limbs or muscle mass is important, while also having important practical ramifications (MacDonald et al., 2000; de Almeida et al., 2010). Many people exercise with their arms by choice for sport (kayakers) or for job requirements (laborers), while others are forced to due to other circumstances (paraplegia, orthopedic limitations). Gaining a better understanding of the potential cardiovascular benefits of such exercise becomes important in optimizing health and performance for such individuals.

Therefore, the purpose of the current study was to determine the roles of exercise intensity (endurance vs. interval training) and limb dependency (legs vs. arms) on the hemodynamic responses during exercise and especially during recovery (< 24 h). We hypothesized that: (i) Endurance exercise performed with the lower body (cycling), and brief Intervals performed with the lower body (Intervals_Legs_), would elicit similar PEH and subsequent increases in blood volume despite the five-fold difference in exercise volume completed, and; (ii) Intervals_Legs_ would elicit larger PEH and subsequent hypervolemia compared to intervals performed with the upper body (Intervals_Arms_) when performed at matched relative exertion (maximal voluntary intensity). As a secondary purpose, we examined Troponin T responses—as a specific marker of cardiac “damage”—to the disparate exercise profiles. Plasma concentrations of this marker rise in healthy individuals following both prolonged endurance exercise (e.g., a marathon) and intense endurance exercise (30-min run) (Shave et al., 2005, 2010; Middleton et al., 2008). Although the Troponin release is thought to be physiological rather than pathological in exercise, little is known about the effect of different exercise parameters (intensity, duration, type) on its release. This study was therefore an opportunity to examine Troponin T release in healthy participants following unaccustomed exercise, as a measure of the cardiac stress involved in interval training.

## Methods

### Participants

Twelve untrained males (mean ± SD; age: 23 ± 3 years; body mass: 72.4 ± 12.8 kg, height: 174 ± 8 cm, BMI: 24.0 ± 3.9 kg·m^−2^, maximal aerobic power: $\overset{\cdot }{\text{V}}$O_2_ max: 44 ± 8 mL·kg^−1^·min^−1^) were recruited to the study, which was approved by the University of Otago Human Ethics Committee and conformed to the standards set by the Declaration of Helsinki. Untrained was classified as undertaking two or fewer 30-min exercise sessions per week in the previous 6 months. All participants were non-smokers and abstained from caffeine and alcohol for 24 h before, and throughout, each experimental trial. Participants were informed of the experimental procedures and possible risks before giving written informed consent.

### Experimental protocol

#### Design

A Latin Square study design was used with a balanced crossover of conditions. All trials were at the same time of day and separated by an average of 2 weeks (range: 8–30 days) to ensure adequate wash out. Participants underwent a familiarization session, in which initial screening was completed, and an incremental exercise test to exhaustion to determine their cycling $\overset{\cdot }{\text{V}}$O_2_ max (see below). The Endurance exercise was set at a workload corresponding to 65% $\overset{\cdot }{\text{V}}$O_2_ max. Participants also conducted one 30-s interval for each of the Interval conditions to familiarize with the Intervals_Legs_ and Intervals_Arms_ protocols. In a following session, blood volume was measured using carbon monoxide dilution (Schmidt and Prommer, 2005), at least 1 week following $\overset{\cdot }{\text{V}}$O_2_ max determination and within 1 week before their first trial.

Participants' diet was standardized (i.e., provided to them) over the 15 h before and 22 h during each trial because protein intake is known to affect albumin synthesis and catabolism, and therefore plasma volume expansion (de Feo et al., 1992; Okazaki et al., 2009a,b). Participants were advised to avoid physical activity in the 24 h before and during each experimental trial (monitored using physical activity recall logs).

*The* $\overset{\cdot }{V}$*O*_2_ *max test*, undertaken to characterize the cohort and establish workrate for Endurance exercise, was undertaken on an electromagnetically-braked cycle ergometer (Velotron, RacerMate Inc, Seattle, USA). Participants warmed up for 5 min at a self-selected workrate, then undertook the following incremental protocol to exhaustion: Workrate started at 100 W and incremented by 33 W at 3-min intervals for four increments before ramping upward at 25 W per minute until voluntary exhaustion. Respiratory gases were sampled breath-by-breath (Cosmed Cardio Pulmonary Testing, CosmedSrl, Rome, Italy), and $\overset{\cdot }{\text{V}}$O_2_ max was calculated from the highest 30-s averaged value. The Endurance exercise workrate was then determined by interpolating the steady-state $\overset{\cdot }{\text{V}}$O_2_-to-workrate relation established from the first four stages of the test.

#### Procedure for each trial

**Figure 1:**
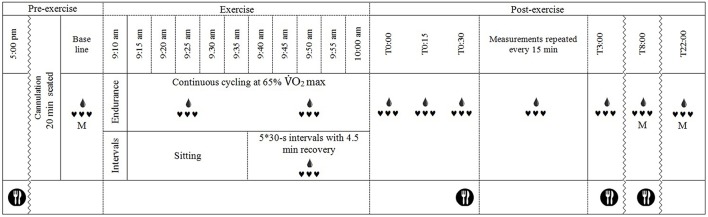
**Schematic of the experimental timeline**. 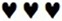, continuous heart rate and beat-to-beat blood pressure recordings, M, manual blood pressure measurement; 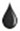, blood sample drawn (every 15 min after exercise except at T2:45); and 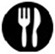, standardized meal fed to participants.

A schematic of the basic testing procedure is shown in Figure 1. Participants arrived at the laboratory at 8:00 a.m. in a fasted state and were asked to void their bladder before height, body mass and urine specific gravity were measured. Thereafter, a flexible cannula was inserted into a suitable vein in the antecubital fossa (for Endurance and Intervals_Legs_ trials) or the distal forearm (for Intervals_Arms_). Participants sat in a standardized chair for 20 min before beginning baseline measurements, during which time a photoplethysmographic cuff was fitted on the index finger and a manual (auscultatory) cuff on the proximal segment of the non-cannulated arm. Baseline measurements were for 10-min; consisting of beat-to-beat blood pressure for 8 min via photoplethysmography (see Measurements), 1 min of manual blood pressure recordings, then a blood sample during the 10th min. Exercise then commenced at 09:10 a.m. for Endurance, and 09:35 a.m. for Interval sessions, such that recovery from exercise began at 10:00 a.m. for all sessions.

*The exercise protocols* were near replications of those described by Burgomaster et al. (2008). The Endurance protocol consisted of 50 min cycling at 65% $\overset{\cdot }{\text{V}}$O_2_ max on a cycle ergometer (Velotron, RacerMateInc, Seattle, USA). $\overset{\cdot }{\text{V}}$O_2_ was analyzed (Cosmed Cardio Pulmonary Testing, CosmedSrl, Rome, Italy) during the initial 5 min and work load adjusted if necessary to ensure that 65% $\overset{\cdot }{\text{V}}$O_2_ max was achieved; work load was fixed thereafter. Both interval protocols comprised 5 × 30-s maximal intervals separated by 4.5 min of active recovery. The Intervals_Legs_ were performed on the same ergometer as Endurance, against a resistance of 0.075 kg·kg body mass^−1^. The Intervals_Arms_ protocol was performed on the same ergometer with a custom adaptation for arm-cranking, and was against a resistance 0.0375 kg·kg body mass^−1^. Participants were verbally encouraged to continue pedaling/cranking as fast as possible throughout each interval to achieve maximal effort. During the 4.5-min recovery period between intervals, participants remained on the bike (or seated for arm crank) and were permitted to cycle at a low cadence against light resistance (30 W for Intervals_Legs_ and 15 W for Intervals_Arms_) to limit venous pooling, sensations of light-headedness or nausea (Burgomaster et al., 2008). Exercise was terminated if participants showed signs of distress or inappropriate physiological responses (e.g., vomiting or severe nausea). Air velocity was 2.2 m·s^−1^, generated using a large-diameter fan, and the exercise and rest were in temperate conditions (21 ± 1°C, 45 ± 7% relative humidity) to limit effects of heat stress on PEH (Halliwill et al., 2013).

*For recovery*, within 1 min of finishing exercise, participants were moved to a chair (within 1 m of ergometer) and seated in a standardized position, in which they remained for 3 h. Following 3 h, participants were allowed to leave the laboratory and undertake their daily routine, without any strenuous physical activity (i.e., necessary walking for normal daily activities (e.g., to get to lectures) was permitted). Participants then returned to the laboratory at 18:00 h that evening (post prandial) and 08:00 the next morning (fasted) for further measurements of blood pressure (auscultatory) and blood constituent concentrations following 20-min seated rest. Dinner and breakfast, respectively, were supplied following these measurement periods.

### Measurements

*Blood pressure* was measured beat-to-beat using finger photoplethysmography (Finometer, Finapress Medical Systems, The Netherlands) at baseline and throughout the initial 3 h following exercise. The participant's hand rested on the armrest of a chair for all measurements (including 8 and 22 h post-exercise measures). Blood pressure was recorded for 5 min every 15 min in this 3-h period, then averaged across the middle 3 min. Photoplethysmography data were sampled at 200 Hz and converted to digital recordings (PowerLab/16SP ML795; ADInstruments, Dunedin, New Zealand) for storage to computer and analysis offline (Chart v7.3, ADInstruments). Auscultatory measurements were made using Korotkoff sounds (Pickering et al., 2005) at baseline, and 8 and 22 h following exercise. Measurement was in duplicate, 1 min apart, by the same experienced operator, using an aneroid sphygmomanometer (certified desk type, CE 0483, China) and stethoscope (Littmann Classic II, USA).

#### Blood constituents

Venous blood samples (4 mL) were collected, without stasis, through the cannula (22 gauge, Teflon) into tubes containing EDTA-anti-coagulant, at baseline, immediately after exercise and at each 15-min interval throughout the initial 3 h following exercise. At 8 and 22 h following exercise, venous samples were obtained via venapuncture, without stasis. Hematocrit and hemoglobin concentration were measured from each sample immediately for calculation of changes in plasma volume, using the method outlined by Strauss et al. (1951). Hematocrit was measured from duplicate 75-μL capillary tubes, after centrifugation at 3500 rpm for 10 min (IEC Micro CL 17 centrifuge, Germany), to an accuracy of 0.1 mm, using metric calipers. Hematocrit was corrected for trapped plasma and whole-body hematocrit (Chaplin and Mollison, 1952; Chaplin et al., 1953). The hemoglobin concentration was measured in duplicate using the modified methemoglobin reaction method (HemoCue Hb 201+, Ängelholm, Sweden). Remaining blood was immediately centrifuged at 4°C, after which plasma was stored at −80°C for later batch analysis of albumin, total protein, Troponin T and osmolality. Plasma albumin and total protein at baseline, 0, 3, 8 and 22 h following exercise were analyzed in duplicate using automated spectrophotometry (Cobas c 111, Roche Instrument Centre, Switzerland). Plasma Troponin T (TnT) was analyzed at baseline, 0, 3, 8, and 22 h following exercise using the Roche high-sensitivity assay (Cobas 6000 Analyzer, Roche Diagnostics GmbH, Mannheim), the basis of which is an electrochemilunescence immunoassay using two monoclonal antibodies specific for cardiac TnT. The upper reference limit for this assay is 14 ng·L^−1^, as this represents the 99th centile of a healthy population (Thygesen et al., 2012), so ≥14 ng·L^−1^ is considered abnormal. Plasma osmolality was analyzed at baseline, 0, 8 and 22 h following exercise using vapor pressure osmometry (Vapro® 5520, Wescor, USA). Samples (10 μL) were measured in duplicate or triplicate (if first two samples >3 mmol·kg^−1^ apart).

### Data analysis

Blood pressure and plasma volume responses were modeled for key parameters (e.g., nadir) before inferential analyses using contemporary recommendations (Hopkins et al., 2009). Area under the curve (AUC), as one such parameter for PEH and hypovolemia, was calculated using the AUC_*G*_ method outlined by Pruessner et al. (2003). The response parameters were used to answer the two distinct questions—effect of exercise type (i.e., Endurance vs. Intervals_Legs_) and of exercise limb (Intervals_Legs_ vs. Intervals_Arms_)-using available spreadsheets (xPrePostCrossover.xls, at www.newstats.org). In determining the smallest clinical change thresholds for meaningful response magnitudes, a 1% change was used for plasma volume change (Convertino, 1983) and a 2 mm Hg change for arterial blood pressure (Cook et al., 1995). For measurements with an unknown smallest clinical change threshold, the default value of 0.20 Cohen units was used. The probability that an outcome effect is positive, trivial or negative refers to whether it is clinically, practically or mechanistically meaningful (positive or negative) or not (trivial) (Hopkins, 2006). Comparisons of major interest are reported as mean differences with 95% confidence intervals, corrected to preclude trivial effects as described above. Paired *t*-tests were used for simple comparison between two experimental conditions (e.g., AUC), and two-way repeated measures ANOVA for time-dependent data (e.g., osmolality change). The ANOVAs were corrected for violations of sphericity using the Huynh-Feldt correction, and post-hoc comparisons were undertaken using the Bonferroni correction, using α = 0.05. Linear regression was used to explore the relation between the 22-h hypervolemic responses and the preceding PEH.

## Results

All participants completed all familiarization, standardization and experimental procedures, except that Intervals exercise was truncated in 3 of 24 trials due to nausea caused by the intense exercise. Trials were separated by at least 8 days (normally 14) within participants. Results are given for the first research question (effect of exercise type; Endurance vs. Intervals_Legs_) before the second question (effect of limbs used; Intervals_Legs_ vs. Intervals_Arms_).

### Blood pressure

#### Exercise type

**Figure 2:**
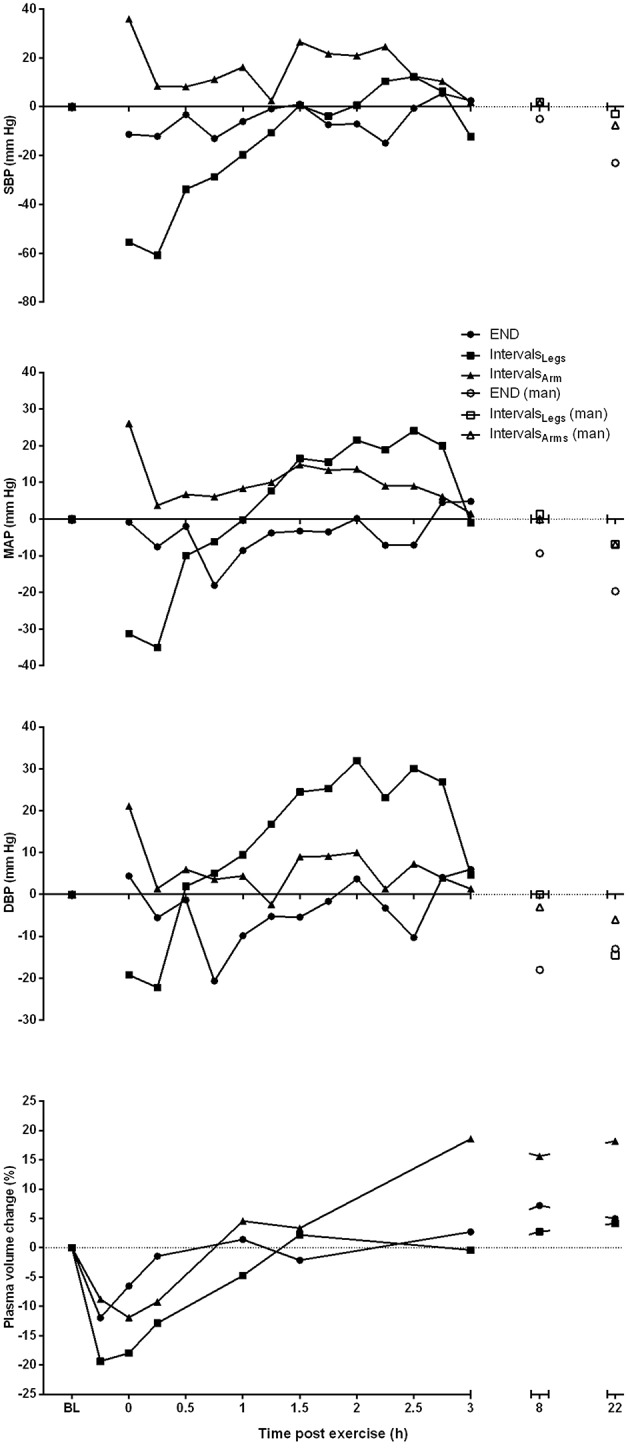
**Profile of change from pre-exercise baseline for systolic (SBP), mean arterial (MAP) and diastolic (DBP) blood pressure, and plasma volume for one participant, across the period from baseline (BL) through to 22 h following each of the three different exercise conditions**. Blood pressure measures taken at 8 and 22 h are manual (man) recordings.

**Figure 3:**
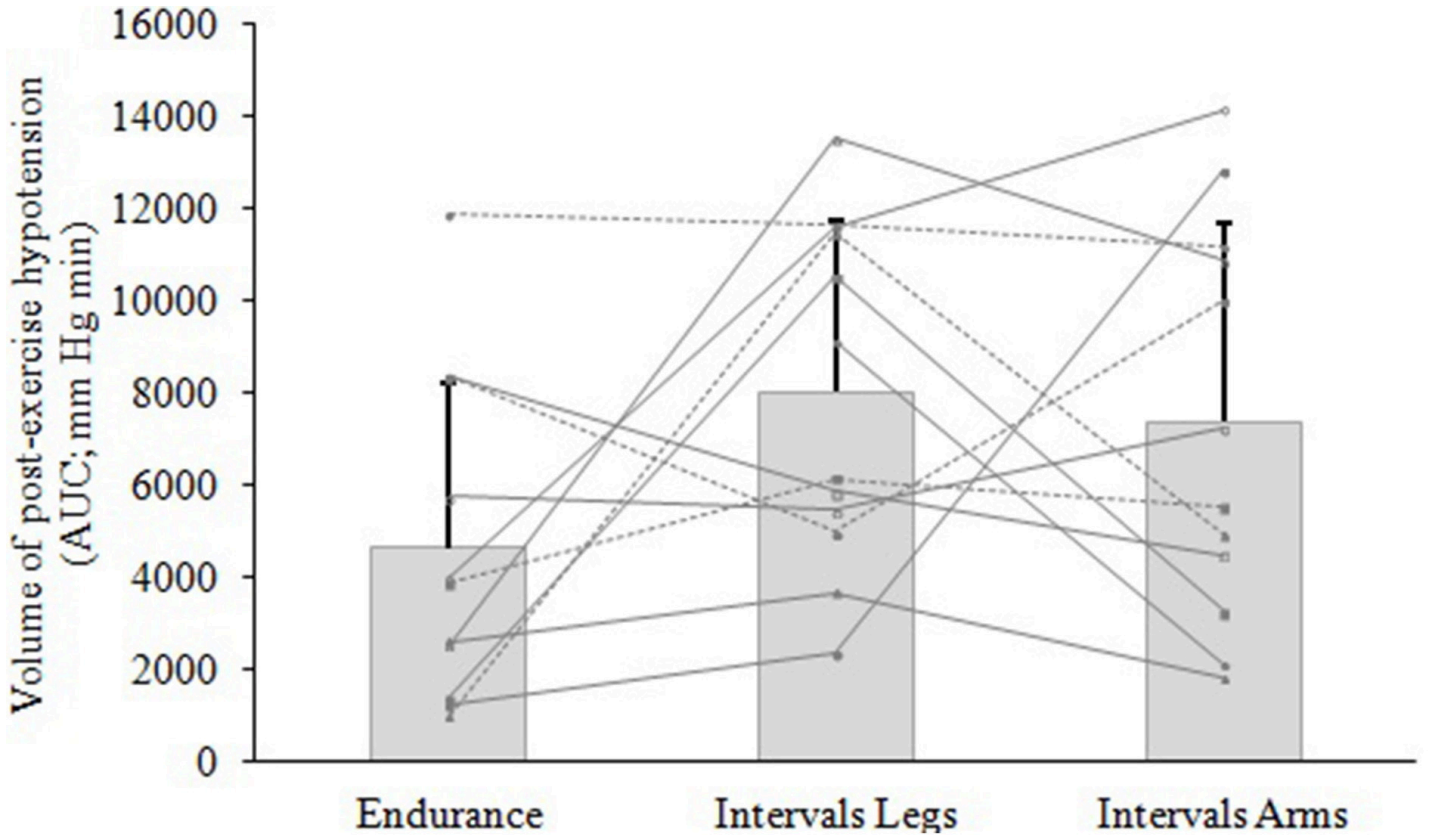
**Volume of hypotension following exercise of different type (Endurance, Intervals_**Legs**_) or different limbs used (Intervals_**Legs or Arms**_)**. Data are mean ± SD (bars) and individual responses (lines) of area under the curve (AUC) for the change in mean arterial pressure (MAP), for 12 participants. AUC is calculated from immediately following exercise until MAP returned through baseline.

**Figure 4:**
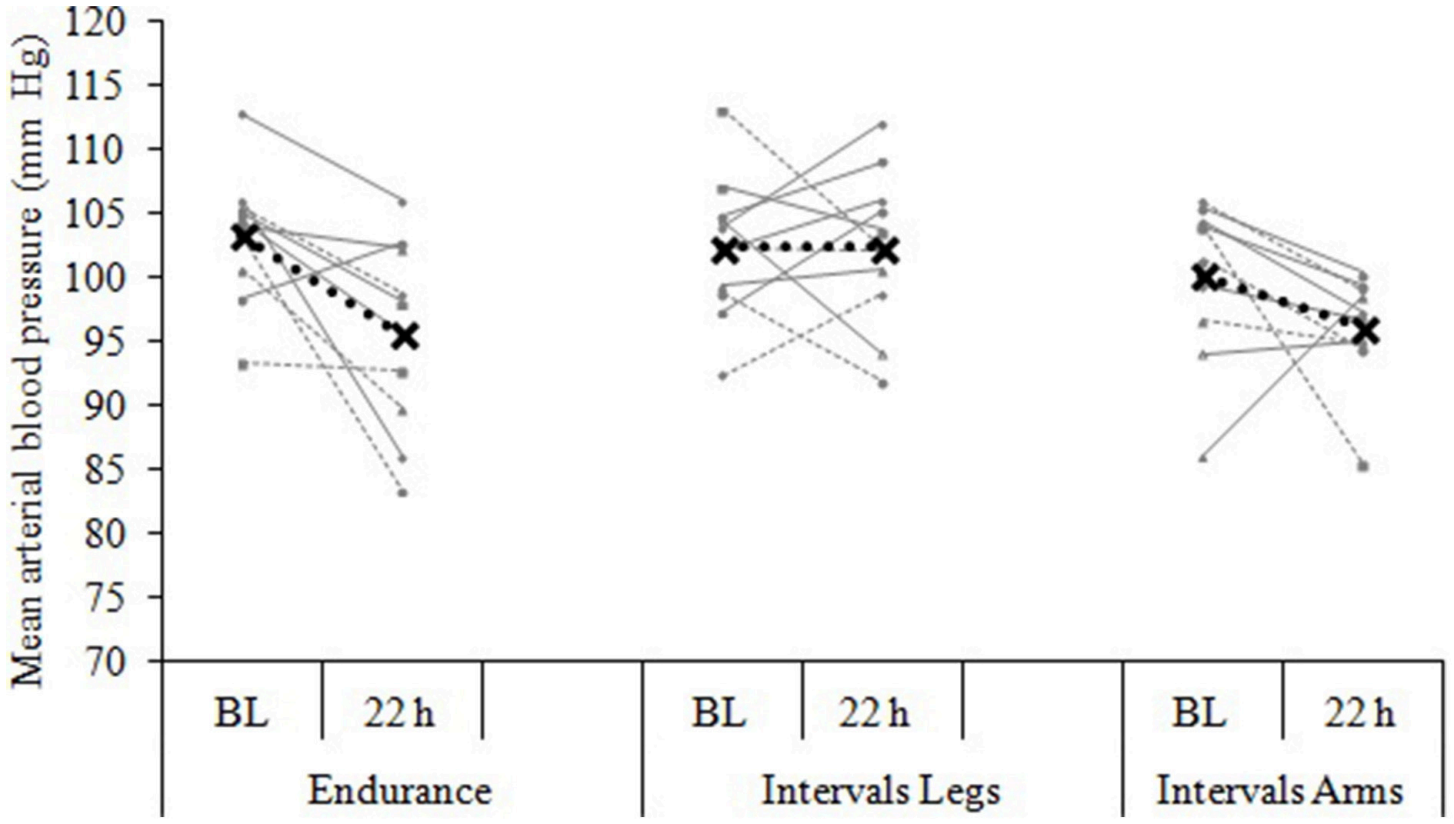
**Blood pressure change from baseline (BL) to 22 h following each of three exercise conditions**. Data are the individual response (gray lines) and mean (black dashed line) of 12 participants, with all measures taken at the same time of day, in a standardized seated position.

The mean blood pressure profiles for different forms of exercise are shown in Table 1, and a typical profile of blood pressure (and plasma volume) responses is shown in Figure 2. The MAP was similar at baseline between Endurance and Intervals_Legs_ conditions (*p* = 0.68). The nadir of PEH occurred at similar time between exercise types (*p* = 0.88), and while there were on average differences for the hypotension magnitude and its duration (time to re-attain baseline), these were not statistically reliable (*p*≥0.26, see Table 1). However, the volume of PEH (AUC) was almost twice as large for Intervals_Legs_ as for Endurance (Table 1, and Figure 3 for individual responses; *p* = 0.05). The probability that this difference was larger/trivial/smaller than for Endurance was 98/0/2%, respectively. On the day after exercise (22 h), MAP was 8 ± 8 mm Hg lower than baseline for Endurance (*p* = 0.01; Figure 4), while Intervals_Legs_ showed no reliable change from baseline (0 ± 7 mm Hg; *p* = 0.99). This 8 mm Hg difference between conditions was significant (*p* = 0.04, CI: 8 ± 7 mm Hg), with the chance of the true effect for Endurance being beneficial/trivial/adverse as 95/4/1%, respectively.

**Table 1:** **Parameters of mean arterial pressure and plasma volume responses following endurance and interval exercise conditions**.

	**Endurance**	**Intervals_Legs_**	**Intervals_Arms_**	**Mean (95%CI) for Intervals-Endurance**	**Mean (95%CI) for Legs-Arms**
**MEAN ARTERIAL PRESSURE**					
Baseline (mm Hg)	103 (5)	102 (5)	100 (4)		
PEH nadir (h:min)	1:05 (0:59)	1:01 (0:55)	0:28 (0:35)	−0:06 (0:54)	0:36 (0:12)
PEH amplitude (mm Hg)	−17 (12)	−22 (9)	−14 (16)	−5 (10)	−8 (11)
PEH duration (h:min)	1:11 (1:43)	1:53 (0:58)	1:37 (1:39)	−0:42 (1:36)	0:18 (1:12)
PEH AUC (mm Hg·min)	3897 (2757)	7540 (3853)	6420 (3947)	3392 (3372)	−1261 (3896)
**PLASMA VOLUME**					
Hypovolemia nadir (h:min)	0:13 (0:06)	0:18 (0:04)	0:21 (0:06)	−0:05 (0:04)	−0:03 (0:04)
Hypovolemia peak amplitude (%)	12 (6)	18 (3)	21 (6)	−6 (4)	−3 (4)
Hypovolemia duration (h:min)	0:15 (0:13)	1:11 (1:24)	0:49 (0:25)	0:54 (0:54)	−0:24 (0:54)
AUC (%·min)	251 (175)	789 (268)	692 (284)	537 (236)	−97 (237)

#### Exercised limbs

At baseline, MAP was similar between the two Interval conditions (*p* = 0.53). On average, the nadir of the PEH occurred ~30 min earlier following intervals with arms than legs, but this difference was not significant (*p* = 0.12). The amplitude (*p* = 0.14) and duration (*p* = 0.61) of hypotension were also not reliably different between conditions, and nor was its volume (AUC; Figure 3; *p* = 0.48, CI for difference: −5157 to 2635 mm Hg·min). The change in MAP from baseline to 22 h also showed an unclear effect between Intervals_Legs_ and Intervals_Arms_ (*p* = 0.20, CI: −4 ± 7 mm Hg, Figure 4).

### Plasma volume

#### Exercise type

**Figure 5:**
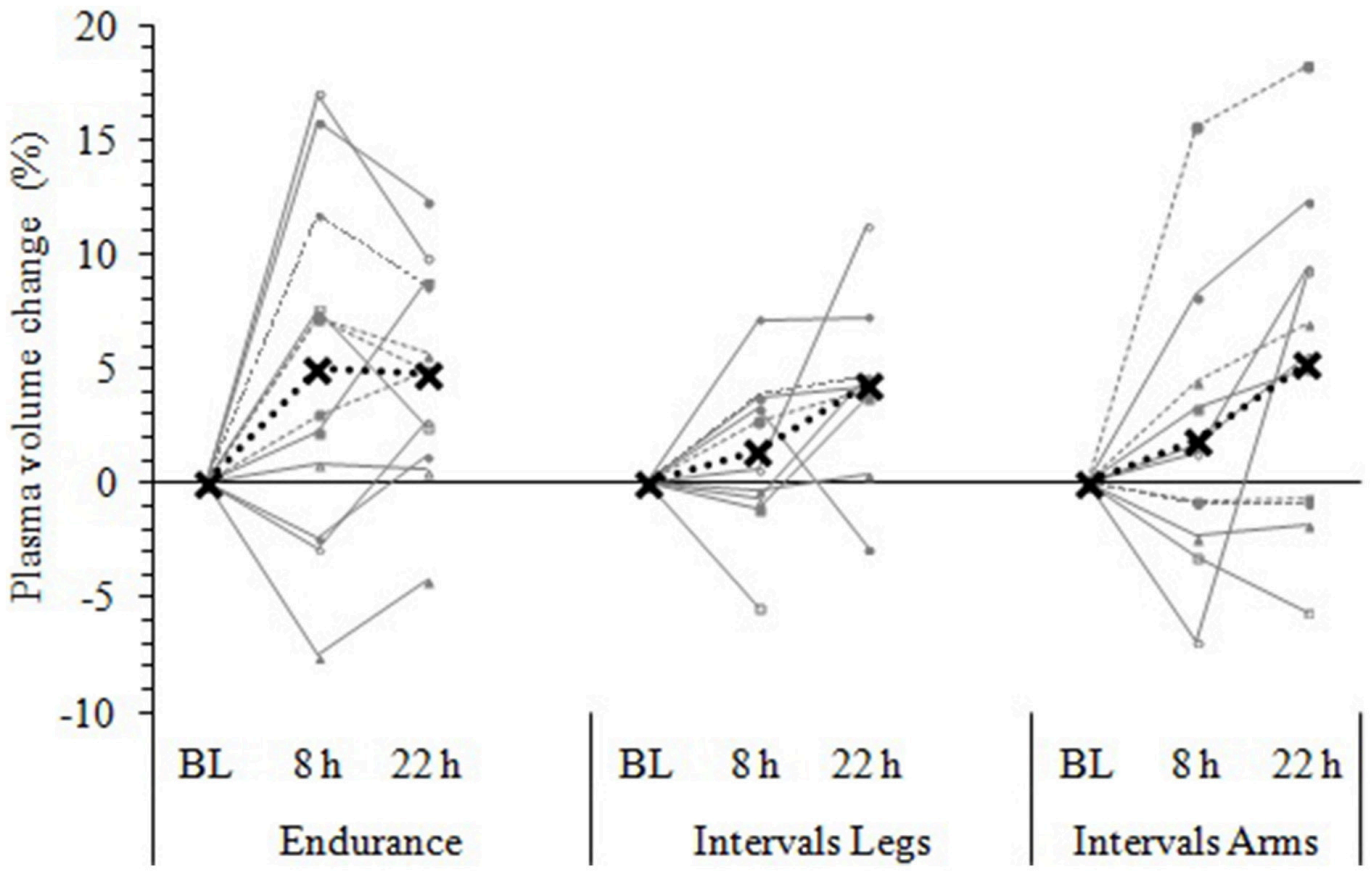
**Change in plasma volume from baseline (BL) to 8 and 22 h following each exercise condition**. Data are the individual response (gray lines) and mean (black dashed line) of 12 participants, with all measures taken at the same time of day, in a standardized seated position, while fasted (BL and 22 h) or post prandial (8 h). Plasma volume change was measured via the hemoglobin-hematocrit method.

Following exercise, the amplitude of hypovolemia was 50% larger following Intervals_Legs_ than Endurance (*p* = 0.02, Table 1). Plasma volume returned through baseline earlier following Endurance (*p* = 0.04), so the volume of post-exercise hypovolemia (AUC) was ~3 fold larger for Intervals (*p* < 0.001); the chance of this difference being positive/trivial/ negative was 100/0/0%, respectively. By the following day, plasma volume had increased by 5 ± 5% for both modes of exercise (between condition effect, *p* = 0.87). However, the progression of this rebound hypervolemia may have differed between conditions (see Figure 5).

#### Exercised limbs

Immediately following exercise the amplitude of hypovolemia did not differ reliably for Intervals performed with the Legs versus Arms (*p* = 0.12, Table 1). Neither did its duration (*p* = 0.38) or volume (AUC: *p* = 0.38). By 22 h, plasma volume had increased 5 ± 7% above baseline following Intervals_Arms_ (Figure 5), which was comparable (*p* = 0.68) to that following Intervals_Legs_.

**Figure 6:**
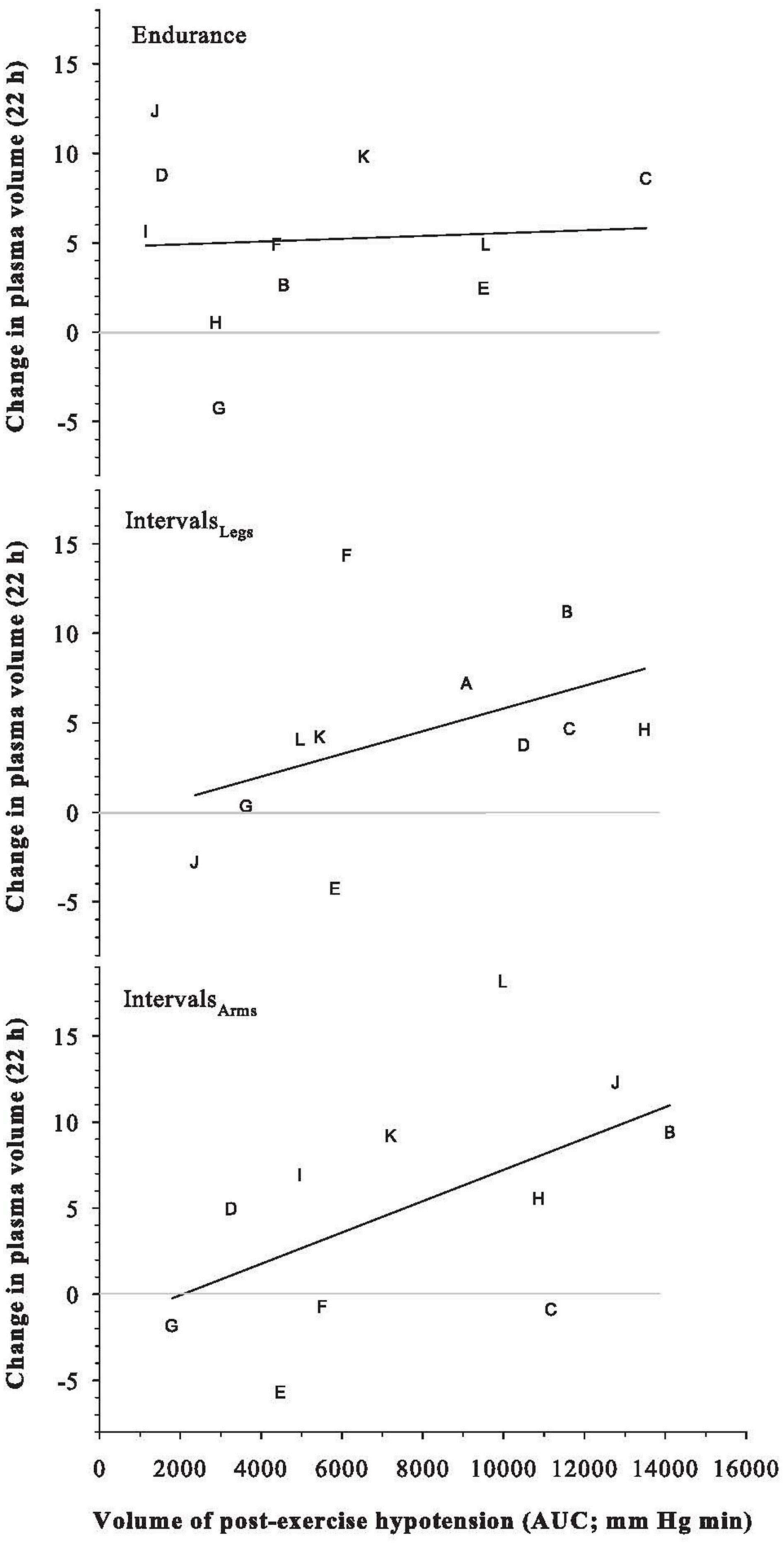
**Relation between the Change in Plasma Volume at 22 h after exercise, and the volume of the preceding post-exercise hypotension**. Symbols are individual responses and the line is the corresponding linear regression. See text for further detail.

Plasma, red cell and blood volumes at rest were 47.7 mL·kg^−1^ (SD: 4.7), 33.3 mL·kg^−1^ (SD: 3.8), and 80.9 mL·kg^−1^ (SD: 7.4), respectively. These absolute plasma and blood volumes were not predictive of the change in plasma volume following any exercise trial (all *r*^2^ ≤ 0.19). Participants' maximal aerobic power was similarly not predictive of their change in plasma volume (*r* = −0.33 for Endurance, −0.21 for Intervals_Legs_, and −0.24 for Intervals_Arms_). As shown in Figure 6, the magnitude of PEH (AUC of mean arterial pressure) was modestly predictive of the change in plasma volume following Interval exercise (Intervals_Legs_: *r*^2^ = 0.19; Intervals_Legs_: *r*^2^ = 0.29), for which the threshold PEH in this cohort appeared to be an AUC of ~2000 mm Hg min. No such relation between PEH and change in plasma volume was evident following Endurance exercise (*r*^2^ = 0.004).

### Plasma solutes (Table 2)

#### Exercise type

The time profiles of solute responses to exercise are shown in Table 2. All solutes showed elevated concentrations immediately after exercise (all *p* < 0.01), but osmolality increased more across Interval than Endurance exercise, by 14 mOsmol·kg^−1^ (interaction: *p* < 0.01; CI: 6 to 21). Protein concentrations had recovered within 3 h following both types of exercise, whereas plasma osmolality showed more prolonged elevation following Intervals_Legs_, which at 22 h, reflected less variability between individuals.

**Table 2:** **Plasma total protein and albumin concentrations before exercise (Baseline) and absolute change scores at 0, 8, and 22 h following exercise**.

	**Baseline**	**Δ @ 0 h**	**Δ @ 3 h**	**Δ @ 8 h**	**Δ @ 22 h**
**PLASMA TOTAL PROTEIN (g**·**L**^−1^**)**					
Endurance	73 (6)	7 (3)^*^	2 (6)	4 (7)	−1 (3)
Intervals_Legs_	69 (3)	11 (2)^*^	1 (3)	3 (3)	1 (2)
Intervals_Arms_	70 (4)	12 (2)^*^	3 (3)	2 (8)	2 (4)
**PLASMA ALBUMIN (g**·**L**^−1^**)**					
Endurance	48 (4)	4 (2)^*^	1 (4)	2 (3)	0 (2)
Intervals_Legs_	46 (1)	6 (1)^*^	1 (1)	2 (2)	1 (1)
Intervals_Arms_	46 (2)	7 (1)^*^	2 (2)	2 (4)	1 (2)
**PLASMA OSMOLALITY (mOsmol**·**kg**^−1^**)**					
Endurance	282 (14)	6 (7)	—	2 (8)	8 (11)
Intervals_Legs_	286 (4)	20 (10)^*^	—	8 (7)^*^	5 (4)^*^
Intervals_Arms_	287 (6)	14 (6)^*^	—	4 (11)^*^	4 (9)

*Data are mean (SD) protein concentrations for n = 11, and osmolality for n = 10*.

* *Denotes p < 0.05 from baseline*.

Plasma cardiac TnT was elevated (>14 ng·L^−1^) in 5/12 participants within the 22-h study period after Endurance; in the range of 16 – 70 ng·L^−1^, while no participant showed elevated TnT after Intervals_Legs_. The mean difference in peak TnT between Endurance and Intervals_Legs_ was 11 ± 13 ng·L^−1^. The AUC of TnT elevation across the 22 h following exercise was larger (by 5843 ng·L^−1^·min) for Endurance than for Intervals_Legs_ (*p* = 0.04; CI: 11476–209).

#### Exercise limbs

Interval exercise using the legs or arms had comparable effects on the mean changes in total protein (*p* = 0.39) and albumin (*p* = 0.44) concentrations, both acutely and at 22 h following exercise (Table 2). Plasma osmolality increased acutely by an average of 14 mOsmol·kg^−1^ with Intervals_Arms_, which was two-thirds of the increase with Intervals_Legs_, but this was not a reliable difference (*p* = 0.11, CI: −6 ± 7 mOsmol·kg^−1^; chances of more/trivial/less difference 3/8/89%, respectively).

Plasma concentration of cardiac TnT was not elevated (>14 ng·L^−1^) in any participant following Interval exercise of either the legs or arms. The mean difference in peak TnT between Intervals_Legs_ and Intervals_Arms_ was 2 ± 2 ng·L^−1^ (*p* = 0.16). The TnT AUC across the 22 h following exercise did not differ according to which limbs were exercised (mean difference 634 ng·L^−1^·min; *p* = 0.24; CI: −1749 – 481). All TnT results were normal (< 14 ng/L) by 22 h.

## Discussion

The main aim of the current study was to investigate the roles of exercise-intensity profile and exercised limbs on hemodynamics acutely (< 24 h), because effects within this timeframe underpin both the adaptive responses and the daily dosage benefits of exercise. The main findings were that: (1) The volumes (AUC) of PEH and hypovolemia were more pronounced following brief maximal-effort exercise Intervals than Endurance exercise, despite much lower net duration of Intervals; (2) whereas, at 22 h after exercise, MAP was unchanged from baseline for Intervals but lowered for Endurance; (3) both forms of exercise stimulated plasma volume to expand by 5% in the first 22 h, and; (4) the hemodynamic responses to Intervals were independent of whether it was performed with the arms or legs, despite their different muscle mass (and vascular capacity) and gravitational dependency. These findings therefore show different hypotensive profiles to the pattern of exercise but not to the limbs used, and show that similar improvements in blood (plasma) volume may be obtainable via a wide variety of moderate-to-severe intensity exercise with different muscle groups.

While blood pressure change and plasma volume expansion are discussed here as separate entities it is clear that they are interdependent. For example, at least some reduction in MAP following exercise appears to be a prerequisite stimulus to plasma volume expansion (Nagashima et al., 1999; Hayes et al., 2000), whereas during exercise the increase in ultrafiltration due to increased MAP and capillary hydrostatic pressure causes plasma volume to decrease and thereby has some effect on blood pressure (Holtzhausen and Noakes, 1995). The effects of exercise intensity and of exercised limbs are discussed concurrently here because that is more useful when considering the causation of PEH, early stage hypovolemia and rebound hypervolemia.

### Blood pressure

The current results indicate that while some specific parameters of the PEH are comparable for vastly different forms of exercise, the magnitude (AUC) seems much more dependent on the exercise intensity (and perhaps the consequently intermittent profile) than on its duration. The potency of high-intensity exercise is emphasized in that a total of just 2.5 min of maximal-effort exercise has more effect than a “typical” endurance-intensity exercise bout of ~5 times the energy usage and ~10 times the net duration (~2 times the gross duration). While others have found comparable rather than larger effects of such Intervals when compared with sustained exercise at moderate intensity (Rossow et al., 2010; Lacombe et al., 2011), all findings support the basic notion that sprint-interval exercise impacts PEH disproportionately to the additional intensity.

We observed similar magnitudes of acute PEH between upper- and lower-body ergometry at matched relative (maximal-effort) workloads. Similar results were obtained by MacDonald et al. (2000) for PEH following 30-min arm ergometry at 65% and leg ergometry at 70% limb-specific $\overset{\cdot }{\text{V}}$O_2_ max in borderline hypertensive individuals, and by de Almeida et al. (2010) for PEH following incremental arm ergometry vs. leg ergometry to exhaustion. The current study adds to the sparse literature on this issue of limb dependency, and shows that the substantive effects on PEH are obtainable from only 2.5 min of net/effective effort.

The comparable changes in PEH after both Intervals_Legs_ and Intervals_Arms_ support Halliwill et al. (2013) in indicating that PEH appears to be mediated at least partly by inhibition of central sympathetic vasoconstrictor output rather than solely through a sustained vasodilation of previously active vasculature. Systemic vascular resistance has been found to be reduced for 1 h following a maximal graded cycling test (Coats et al., 1989). This concurs with evidence that factors within the central nervous system [substance P (neurotransmitter) and GABAergic (major cardiovascular sympathetic inhibitor)] may be contributors to both the exercise pressor response and PEH (Chen and Bonham, 2010). The marked and consistent finding whereby maximal-effort arm exercise elicits PEH as powerfully as that obtained from higher-volume leg exercise reinforces contemporary understandings of PEH. That is, muscle chemoreceptors are thought to act via the NTS to inhibit sympathetically-mediated vascular constriction, which means that PEH is not dependent on the muscle mass or on its gravitational dependency.

At 22 h following Endurance, MAP was on average 8 ± 8 mm Hg lower than at baseline, while Intervals showed no change from baseline irrespective of whether the exercise had been performed with the lower or upper limb (Figure 4). The lack of a decreased MAP at 22 h following Intervals could potentially be due to a longer recovery time course of the sympathetic nervous system due to the higher exercise intensity. Of note, Goto et al. (2003) observed an improvement in endothelial function after moderate-intensity training, which did not change oxidative stress markers, whereas high-intensity training increased oxidative stress with no change in endothelial function. These authors concluded that the impact of exercise on endothelial function may be dependent on the balance between reactive oxygen species, antioxidant defenses, and their impact on nitric oxide availability (Goto et al., 2003). Similarly, less-favorable shear stress profiles (i.e., if more retrograde flow) during the exercise session may have attenuated endothelial function due to increased sympathetic constriction in arterioles within vasculature beds outside of active muscle. Therefore, in the current study the higher exercise intensity may have caused excessive oxidative stress or less favorable shear stress profiles and therefore inhibited improvements in endothelial function at 22 h.

Delayed effects of exercise on arterial pressures are important for healthy and hypertensive individuals, and the current findings add to a conflicting literature on this issue. For example, supportive but also contrasting findings have been shown in regard to effects of both sustainable exercise and sprint interval training. Forjaz et al. (2004) found no reduction in MAP at 22 h following 45 min of cycling at either 30, 50, or 75% $\overset{\cdot }{\text{V}}$O_2_ max in normotensive males and females; nor did Wallace et al. (1999) following 50 min of intermittent walking (5 × 10 min with 3 min rest periods) at 50% $\overset{\cdot }{\text{V}}$O_2_ max in normotensive males and females 22 h following exercise. Whereas, 2 weeks of sprint interval training using a protocol similar to the one used here, led to meaningful reductions in systolic (−6 mm Hg) and diastolic (−9 mm Hg) pressures at 24 h after the final (6th) session of intervals (Whyte et al., 2010). Irrespective, it is worth noting that the more common, clinical-model of high intensity interval training, involving longer and lower-intensity intervals (1–4 min of exercise to 85–95% HR_max_, 1–3 min apart), seems to show more consistent reductions in arterial pressures (Molmen-Hansen et al., 2012; Ronnestad et al., 2014; Wisløff et al., 2015).

### Plasma volume expansion

The 5% expansion of plasma volume that was evident 22 h following both Endurance and Intervals_Legs_ is consistent with those observed (5–7%) in studies from the John B Pierce laboratory, using a more clinically-acceptable/relevant model of interval exercise [i.e., 8 × 4-min intervals at 85% $\overset{\cdot }{\text{V}}$O_2_ max; (Gillen et al., 1994; Haskell et al., 1997; Yang et al., 1998; Nagashima et al., 1999, 2000)]. Surprisingly, we also observed a similar 5% plasma volume expansion with Intervals_Arms_ at 22 h following exercise. While unexpected, the mechanisms by which they may have occurred seem plausible and unsurprising in retrospect. Despite the differences in muscle mass activation, both stimulated similar post-exercise hypovolemia and hypotension [i.e., presumably in response to afferent input from muscle exercise at matched and maximal relative intensity (Miles et al., 1983)]. This similar hypotensive response would presumably facilitate an expansion of plasma volume via increased renal reabsorption of sodium and increased albumin content due to increased return lymphatic return to the circulation and synthesis, and reduced loss of albumin (Gillen et al., 1994; Haskell et al., 1997; Yang et al., 1998; Nagashima et al., 1999, 2000).

However, the causal link between PEH and subsequent expansion of plasma volume (measured at 22 h) is presumably not this straight forward, because the three-fold larger PEH in Interval exercise yielded a hypervolemic response of equivalent magnitude to that from Endurance exercise, and the participants experiencing larger or smaller PEH did not show a correspondingly larger or smaller hypervolemic response. Neither did their hypervolemic response depend on their baseline blood volume or blood pressure. Some independence thus exists between PEH and subsequent plasma volume expansion. In an attempt to explain this, the differences in the time courses of change seem an important factor. A possible mechanism underlying these different time course profiles is AVP activity and its release in response to exercise intensity. Scott et al. (1997) found AVP levels not to be elevated during 50 min of running at 25 or 45% $\overset{\cdot }{\text{V}}$O_2_ max, whereas it was elevated with running at 65% $\overset{\cdot }{\text{V}}$O_2_ max. Based on these findings it is possible that the Interval exercise in the current study stimulated a larger and more prolonged AVP release due to the higher exercise intensity and disruption of homeostasis. Interval exercise led to a smaller expansion in plasma volume (Figure 5) and increased osmolality (Table 2) compared to Endurance at 8 h. These two key mediators of AVP release indicate that AVP levels may have been elevated to a higher level and for longer following Interval exercise. These potentially elevated levels of AVP might also help explain the lack of MAP change from baseline at 22 h for both Interval exercise conditions compared with Endurance, as well as the different trends in plasma volume expansion that seem evident at 22 h. While the half-life of AVP and plasma renin activity are 5 and 20 min respectively (Convertino et al., 1980), it is possible that a prolonged release of these hormones is stimulated due to the greater disruption of homeostasis following Intervals, causing a plasma volume expansion greater than what was measured in the current study past the 22 h time point. Future research could profile a longer period of post-exercise response to address this speculation.

### Troponin T release

These data indicate that TnT may be released in many (but not all) healthy, sedentary individuals following endurance exercise of moderate duration and intensity. The absence of a TnT elevation following repeated 30-s interval exercise was unexpected, but adds support to the notion that duration of exercise may be more consequential than intensity (Shave et al., 2007; Dawson et al., 2008). However, high-intensity exercise (30 min running, 85–90% $\overset{\cdot }{\text{V}}$O_2_ max) of a different profile than utilized in this study has elicited TnI elevation in some participants (Shave et al., 2010), so volume of exercise is perhaps a more relevant parameter. The patterns of release were non-uniform and not able to be related to specific demographics or exercise data. Further research is required to explore the differential responses and the specific parameters of exercise that induce troponin release.

### Limitations

This study is subject to several limitations. Except for the initial 3 h of laboratory-based monitoring, dietary standardization and activity guidelines, in other respects the post-exercise period was carried out in a relatively free-living environment. It is acknowledged that this free-living environment could have potentially influenced the results at the 8 and 22 h measurement times even with the standardizations. However, it was seen as important to include this free living in the current study to increase the robustness of findings to real-world settings. The study was also limited to male participants due to time constraints on the study duration, given that only one trial would be possible within each menstrual cycle. It cannot be assumed that these results apply to females even within the follicular phase, because of the numerous effects of estrogen and progesterone on central and peripheral (renal and vascular) mechanisms of fluid regulation, and on net effects of oxidative stress, for example. Studies that have compared effects of Intervals and/or Endurance exercise in females and males have found no difference in the net PEH in fit individuals, although underlying causation may differ between sexes (Rossow et al., 2010; Cote et al., 2015). Furthermore, the current findings indicate the involvement of the central nervous, anti-diuretic and renin angiotensin systems in the PEH response without the direct measurements of these.

### Perspectives and summary

The results of the current study indicate the importance of exercise intensity in stimulating acute PEH. Of note, performing Intervals with the lower limbs caused similar post-exercise hypotensive and hypovolemic responses to those following Intervals with the upper limbs, emphasizing the integrative causation of PEH, the mechanisms of which have been elucidated by others (e.g., Chen and Bonham, 2010; Halliwill et al., 2013). Furthermore, at 22 h following exercise, MAP had decreased below baseline values for Endurance but not for Intervals_Legs_, whereas the similarities between lower- and upper-limb exercise were still evident (i.e., neither had reliably caused a longer-term hypotension). These findings indicate that the recovery profiles following Intervals may be different, potentially due to prolonged elevation of sympathetic nervous system activity, elevated circulating stress hormones, or autocrine factors suppressing endothelial function. Importantly, all conditions stimulated a similar 5% expansion of plasma volume by 22 h following exercise. These findings collectively allow a better comprehension of the short-term training response to different exercise stimuli, along with providing evidence for the use of a variety of exercise regimes that could be used by different populations (hypertensive, time constrained, paraplegic) to access the cardiovascular health benefits exercise provides. They also indicate that while Intervals have been shown to have very high metabolic value compared to Endurance, the effects of Intervals on long term blood pressure control maybe not as effective, yet this is equivocal (Whyte et al., 2010). Finally, the marked inter-individual variability in recovery responses following these somewhat polarized forms of exercise—whereby some individuals even showed larger PEH to Endurance than Intervals—further highlights the need for personalized rather than blanket prescription of exercise (MacDonald et al., 2000; Rossow et al., 2010; Lacombe et al., 2011; Luttrell and Halliwill, 2015). While this study used exercise profiles known to be effective in improving fitness, for reasons of safety and acceptance (e.g., avoiding nausea) it would be important to avoid starting training at these session volumes.

## Author contributions

All authors contributed meaningfully to the four contribution criteria. Authors taking lead roles in each phase were: JC for conception of the study, obtaining funding and ethics approvals, and supervising the project; MG for extensive planning of the study, participant recruitment, data collection and analysis, and writing the first draft of the manuscript. MF, SS, and EP assisted extensively in data collection, and SL co-supervised the project through all phases.

## Funding

This study received funding support from the School of Physical Education, Sport, and Exercise Sciences and a University of Otago Research Grant.

### Conflict of interest statement

The authors declare that the research was conducted in the absence of any commercial or financial relationships that could be construed as a potential conflict of interest.

## Abbreviations

AUC: Area under the curve
Intervals_Legs_: Sprint intervals performed with the lower body
Intervals_Arms_: Sprint intervals performed with the upper body
TnT: Troponin T
$\dot{\text{V}}$O_2_ max: maximal aerobic power
kg: kilogram
W: watts
mL: milliliters
μL: microliters
mm: millimeter
rpm: revolutions per minute
MAP: mean arterial blood pressure
SBP: systolic arterial blood pressure
DBP: diastolic arterial blood pressure
mOsmol·kg^−1^: milliosmoles per kilogram of water.
